# An ecological and stochastic perspective on persisters resuscitation

**DOI:** 10.1016/j.csbj.2024.12.002

**Published:** 2024-12-06

**Authors:** Tania Alonso-Vásquez, Michele Giovannini, Gian Luigi Garbini, Mikolaj Dziurzynski, Giovanni Bacci, Ester Coppini, Donatella Fibbi, Marco Fondi

**Affiliations:** a Department of Biology, University of Florence, Via Madonna del Piano 6, Sesto Fiorentino, 50019, Italy; b G.I.D.A. SpA, Via Baciacavallo 36, Prato, 59100, Italy

**Keywords:** 46N60, 37N25, Persisters, Microbial interactions, Microbial communities

## Abstract

Resistance, tolerance, and persistence to antibiotics have mainly been studied at the level of a single microbial isolate. However, in recent years it has become evident that microbial interactions play a role in determining the success of antibiotic treatments, in particular by influencing the occurrence of persistence and tolerance within a population. Additionally, the challenge of resuscitation (the capability of a population to revive after antibiotic exposure) and pathogen clearance are strongly linked to the small size of the surviving population and to the presence of fluctuations in cell counts. Indeed, while large population dynamics can be considered deterministic, small populations are influenced by stochastic processes, making their behaviour less predictable. Our study argues that microbe-microbe interactions within a community affect the mode, tempo, and success of persister resuscitation and that these are further influenced by noise. To this aim, we developed a theoretical model of a three-member microbial community and analysed the role of cell-to-cell interactions on pathogen clearance, using both deterministic and stochastic simulations. Our findings highlight the importance of ecological interactions and population size fluctuations (and hence the underlying cellular mechanisms) in determining the resilience of microbial populations following antibiotic treatment.

## Graphical abstract

**Figure:**
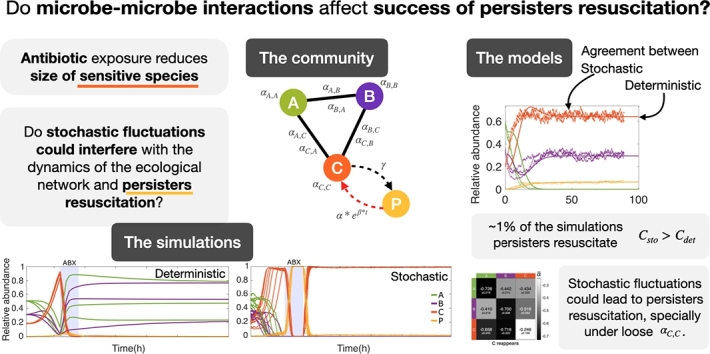

## 1 Introduction

For a long time, antibiotic resistance, tolerance and persistence have been studied from the point of view of single organisms and how each drug could affect their genome, metabolism and growth rate. However, in recent years, the study of microbial communities and how their ecological interactions could alter the efficacy of antibiotics has gained ground. In fact, mounting evidence suggests that such interactions play a crucial role in the emergence and maintenance of antibiotic persistence/tolerance and in the actual efficiency of antibacterial treatments [1], [2], [3], [4], [5], [6]. The work by De Vos et al. (2017), for example, characterised a large set of cooperative and competitive interactions in polymicrobial urinary tract infections, showing that *Proteus mirabilis* predominantly protect some isolates from antibiotics while harmed others in the absence of them. They highlighted the importance of interactions in i) maintaining the stability of the underlying population and ii) affecting the survival of community members during antibiotic exposure [1]. Mizrahi et al. (2023) showed that the biggest benefit usually goes to the more sensitive strains, allowing them to grow in concentrations higher than the minimum for inhibition. Hence, the evolution of antibiotic-resistant and tolerant bacteria can be modified depending on the type and strength of interspecies interactions. Sensitive strains that manage to grow under antibiotic stress tend to have a decrease in the death rate, coupled with a decrease in the growth rate, leading to tolerance; i.e. lower growth rate, more tolerance. However, we need to consider that there must be a slow antibiotic degradation in the environment for the tolerance to evolve [5]. While resistance is inherited and may be acquired by horizontal gene transfer of resistance-encoding genes or mutations, tolerance is the general ability of a population to survive longer to antibiotic treatments by having a lower death and survival rate, without any genetic modifications. There are cells that not only exhibit tolerance to antibiotics but also persist in their presence without undergoing genetic changes. These persister cells are a subpopulation of a growing bacterial population that survives antibiotic treatment by entering a dormant state, which is a reversible, non-inherited phenomenon [7], [8], [9]. Recent evidence suggests that low ATP levels induce protein aggregation and thus non or slow-growing bacterial cells, stochastically favouring persisters formation [10]. Although this low-energy mechanism may be present in several, if not all, bacterial species, this is not the only mechanism that produces persister cells. Toxin-antitoxin (TA) loci, such as the HipA system in *Escherichia coli*, have been implicated in the formation of persister cells [11], [12], [13], [14]. Although its role in this process has been controversial, with conflicting evidence regarding the exact mechanisms involved, HipA might indeed be correlated with persistence [15], [16]. The activation of the bacterial SOS response is yet another mechanism that has been associated with bacterial persistence [17]. Overall, it has been estimated that the fraction of persisters within a microbial population may be typically less than 1-2%, depending on, among other factors, the antibiotics used, the growth phase and the taxonomy of the microbes under study [18], [19], [9], [20], [21], [16]. Once cells have reached a dormancy state of persistence and survived the antibiotic treatment, they may start a resuscitation process: their exit from the persistent state to repopulate, upon cessation of antibiotic exposure [22]. The phenotypic features that allow persister resuscitation are starting to gain attention, since this process may represent an attractive target for antibiotic treatments [23]. Although resuscitation dynamics of a group of persisters has long been considered to be constant over time [24], a recent work suggested that cells resuscitate exponentially rather than with a fixed rate [25]. In 2023, Fang and colleagues modelled the dynamics of resuscitation by single-cell tracking and showed that, in most cases, this does not depend on the treatment duration but only on the antibiotic concentration (tested with ampicillin, imipenem and ciprofloxacin). One essential characteristic that may help the cell is its ability to detoxify the intracellular environment by coupling the inner-membrane ABC transporters with the outer-membrane protein TolC. In other words, changes in the efflux strongly delay the resuscitation process. Interestingly, they observed that persister partitioning is antibiotic-specific. They described two processes that changes the resuscitation of persisters cells: depletion of drug-target binding proteins (cell-wall targeting antibiotics) and the activation of SOS-response (quinolone antibiotics), resulting in triangular structural defects and filamentation/polar partitioning, respectively [26], [27], [25]. This leads to a population formed by healthy, damaged and failed cells (their proportion depending on the antibiotic treatment) that, as a whole, display a reduced growth rate in respect to the pre-antibiotic exposure phase [25].

The problem of resuscitation and, more in general, pathogens clearance, is strongly connected to the small population size of the pathogen species. There are evidences that suggest that, even if the pathogen population has been drastically reduced by the antibiotic treatment, this may not be enough as failure to completely eradicate a population of bacteria may lead to undesired consequences. The reduced population can lead both to i) infection relapse [28], [29] and ii) antibiotic resistance development [30]. Clearly, both scenarios complicate the subsequent treatments. While the dynamics of large populations can be thought of as a deterministic process, the dynamics of small populations may be less predictable and affected by factors that are negligible in the first scenario. The underlying probability of these stochastic processes has been described in different ways [31], [32], [33].

Here, we argue that microbe-microbe interactions in a microbial community represent an additional factor affecting the mode, tempo and success of persisters resuscitation. Since antibiotic exposure drastically reduces the size of sensitive species (virtually to the size of persisters pool), even mildly negative interactions may impede the resuscitation of persisters once their population size has dropped under a certain threshold. At the same time, noise may contribute to this complex scenario, as stochastic fluctuations in cell numbers could interfere with the dynamics of the ecological network and influence the resuscitation of the persister pool. To investigate these issues, we developed a theoretical model of a simple microbial community that includes one pathogenic, antibiotic-sensitive and persisters-generating species together with two other interacting community members and analysed how the interplay among the three players (as well as other model parameters) affects the resuscitation of the persisters, also when considering the role of noise.

## 2 Theory

**Fig. 1:**
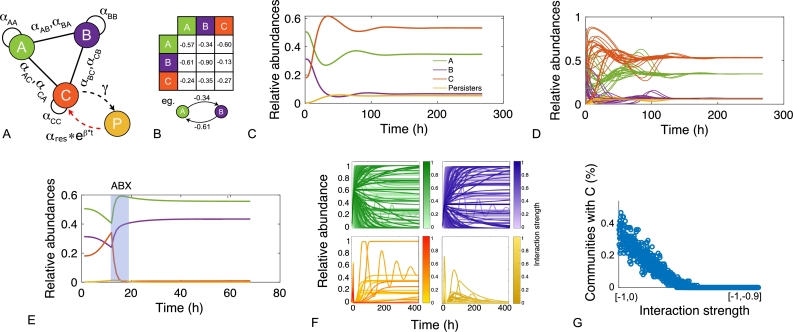
Schematic representation of the three-member community modelled herein. (A) Network describing the dynamics of the species *A* (in green), *B* (in purple) and *C* (in orange), and the possibility of the persister *P* formation (in yellow). (B) Random interaction matrix that produces a stable non-zero steady state. (C) Sample simulation with a final co-existence of all the three species. (D) Dynamics of the community with different initial abundances for each species lead to the same steady state. (E) Dynamics of the community under antibiotic (ABX) stress in the interval 12-18 h. (F) Changes in the final composition of abundances resulting from varying the strength of the interactions among the three community members (i.e. changing the interaction matrix; line opacity indicates interaction strength). (G) Percentage of communities in which species *C* disappears when the interaction strength increases.

In this work, we model a theoretical community made of three interacting species, namely species *A*, *B* and *C* (Fig. 1A). Each member of the community grows according to a specific growth rate (*μ*) and dies according to a specific death rate (*δ*). Species *C* represents a hypothetical pathogen, sensitive to a given antibiotic X (ABX) while *A* and *B* represent two other species that are not sensitive to ABX. Species *A*, *B* and *C* interact with each other and the strength of these interactions is given by coefficients of the type $\alpha_{i,j}$, accounting for the effect of species *i* on species *j* (Fig. 1B). During growth, species *C* generates persisters *P* with a rate *γ*. Persisters *P* do not grow ($\mu_{P}$=0), are not sensitive to antibiotic and do not interact with species *A* and *B*. We then consider the scenario in which the community is exposed to antibiotic for a given amount of time (from $t_{start}$ to $t_{end}$). During this time period, the growth rate of species *C* is set to 0 ($\mu_{C}$=0), an antibiotic killing rate is introduced (*ξ*), all the interactions affecting species *C* are set to 0 ($\alpha_{A,C}$=$\alpha_{C,A}$=$\alpha_{B,C}$=$\alpha_{C,B}$=0) and persisters production rate is set to 0 (*γ*=0). After the exposure to the antibiotic treatment (i.e. when simulation time $t>t_{end}$) we simulate the resuscitation of persisters *P* by converting *P* into *C* with a rate equal to $\alpha_{res}e^{\beta t}$ as reported in [25] for ampicillin treatment in *E. coli* and *Salmonella enterica* and setting back to their original values all the interaction parameters ($\alpha_{i,j}$) and the persisters generation rate (*γ*). As for *C* growth rate after antibiotic exposure, we considered the fact that cells resuscitated from the pool of persisters sometimes manifest a damaged phenotype [25] and set the post-antibiotic growth rate $\mu_{C}$ to the 75% of its original value $\mu_{C}$. Simulations then are continued until all the species have reached their steady state (if any). We used this simple and general modelling framework to evaluate the role of specific parameters (e.g. the duration of the antibiotic treatment, the persisters production rate and, more importantly, the effect of species interactions) on the capability of pathogen species *C* to restore normal growth after the antibiotic-induced perturbation.

The model can be formalised by the following set of equations. We report here the case for species *A*, which is analogous for species *B* and *C*:(1)
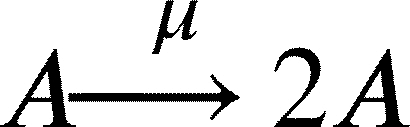
(2)
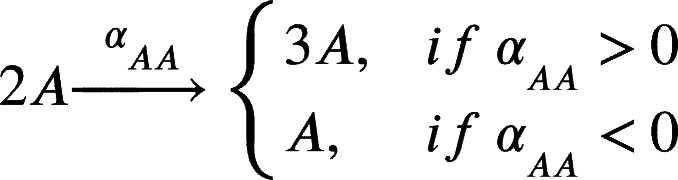
(3)
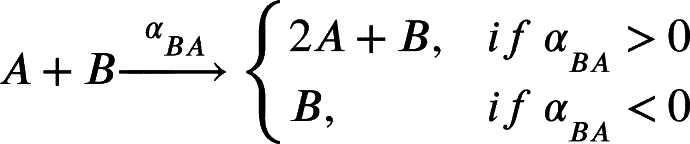
(4)
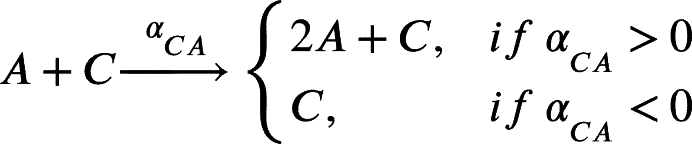
(5)
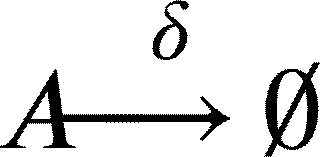


Clearly if any of the above mentioned *α* is equal to 0, no changes in *A* will occur. In particular, for species *C* we also have persisters formation with rate *γ*:(6)
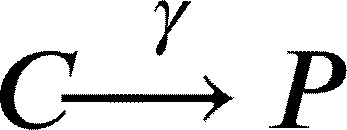


Furthermore, during antibiotic exposure, we include the following equations to the system to account for the antibiotic killing rate *ξ*:(7)
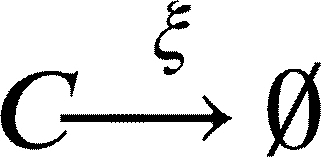


Finally, after the exposure to the antibiotic, we modelled persisters resuscitation as follows:(8)
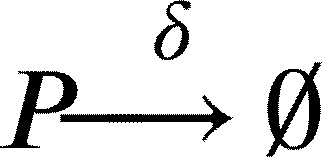
(9)
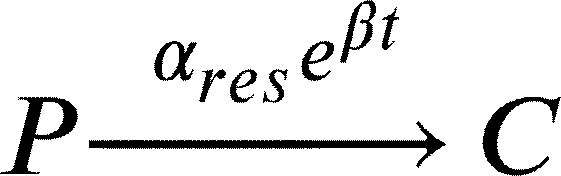


This set of equations leads to the following set of four ordinary differential equations (ODEs):

(10) $$\overset{˙}{\left[A\right]}=\mu [A]+\alpha_{{}_{BA}}[AB]+\alpha_{{}_{CA}}[AC]+\alpha_{{}_{AA}}{\left[A\right]}^{2}-\delta [A]$$

(11) $$\overset{˙}{\left[B\right]}=\mu [B]+\alpha_{{}_{AB}}[BA]+\alpha_{{}_{CB}}[BC]+\alpha_{{}_{BB}}{\left[B\right]}^{2}-\delta [B]$$

(12) $$\overset{˙}{\left[C\right]}=\mu [C]+\alpha_{{}_{AC}}[CA]+\alpha_{{}_{BC}}[CB]+\alpha_{{}_{CC}}{\left[C\right]}^{2}-\delta [C]-\gamma [C]$$

(13) $$\overset{˙}{\left[P\right]}=\gamma [C]-\delta [P]$$

When $t_{start}<t<t_{end}$, i.e. during antibiotic exposure, equation (12) changes to:

(14) $$\overset{˙}{\left[C\right]}=\mu [C]+\alpha_{{}_{AC}}[CA]+\alpha_{{}_{BC}}[CB]+\alpha_{{}_{CC}}{\left[C\right]}^{2}-\delta [C]-\gamma [C]-\xi [C]$$

And when $t>t_{end}$, i.e. after antibiotic exposure, equations (12) and (13) change to:

(15) $$\overset{˙}{\left[C\right]}=\alpha_{res}e^{\beta t}[P]+\rho \mu [C]+\alpha_{{}_{AC}}[CA]+\alpha_{{}_{BC}}[CB]+\alpha_{{}_{CC}}{\left[C\right]}^{2}-\delta [C]-\gamma [C]$$

(16) $$\overset{˙}{\left[P\right]}=\gamma [C]-\delta [P]-\alpha_{res}e^{\beta t}[P]$$

where *ρ* is a scaling factor to account for the growth defect of resuscitated *C* cells. The values of the parameters are reported in Table 1. We also formulated the deterministic model of the community represented by the Eqs. (1) - (9) as an ensemble of stochastic rules in a hypothetical volume (*V*) of 1000 μl, a value that is in line with previous experiments/models on microbial community dynamics [34]. Mathematically, at each time *t* the system is fully described by $\text{x}=(n_{{}_{A}},n_{{}_{B}},n_{{}_{C}},n_{{}_{P}})$, a vector whose components are discrete quantities that identify the total number of elements of species *i* (for i=A,B,C,P). To proceed in the analysis, it is necessary to introduce the transition rates T(y|x) from the state *x* to the new state *y*, where *y* is a state compatible with the equations listed above. These transitions originate from Eqs. (1) - (9) and their explicit forms are as follows.

(17) $$T_{1}(n_{{}_{A}}+1|n_{{}_{A}})=\mu \cdot n_{{}_{A}}$$

(18) $$T_{2}(n_{{}_{B}}+1|n_{{}_{B}})=\mu \cdot n_{{}_{B}}$$

(19) $$T_{3}(n_{{}_{C}}+1|n_{{}_{C}})=\mu \cdot n_{{}_{C}}$$

(20) $$T_{4}(n_{{}_{A}}-1|n_{{}_{A}},n_{{}_{B}})=\frac{\alpha_{{}_{BA}}}{V}\cdot n_{{}_{A}}\cdot n_{{}_{B}},\;if\;\;\alpha_{{}_{BA}}<0$$

(21) $$T_{5}(n_{{}_{A}}+1|n_{{}_{A}},n_{{}_{B}})=\frac{\alpha_{{}_{BA}}}{V}\cdot n_{{}_{A}}\cdot n_{{}_{B}},\;if\;\;\alpha_{{}_{BA}}>0$$

(22) $$T_{6}(n_{{}_{A}}-1|n_{{}_{A}},n_{{}_{C}})=\frac{\alpha_{{}_{CA}}}{V}\cdot n_{{}_{A}}\cdot n_{{}_{C}},\;if\;\;\alpha_{{}_{CA}}<0$$

(23) $$T_{7}(n_{{}_{A}}+1|n_{{}_{A}},n_{{}_{C}})=\frac{\alpha_{{}_{CA}}}{V}\cdot n_{{}_{A}}\cdot n_{{}_{C}},\;if\;\;\alpha_{{}_{CA}}>0$$

(24) $$T_{8}(n_{{}_{B}}-1|n_{{}_{B}},n_{{}_{A}})=\frac{\alpha_{{}_{AB}}}{V}\cdot n_{{}_{B}}\cdot n_{{}_{A}},\;if\;\;\alpha_{{}_{AB}}<0$$

(25) $$T_{9}(n_{{}_{B}}+1|n_{{}_{B}},n_{{}_{A}})=\frac{\alpha_{{}_{AB}}}{V}\cdot n_{{}_{B}}\cdot n_{{}_{A}},\;if\;\;\alpha_{{}_{AB}}>0$$

(26) $$T_{10}(n_{{}_{B}}-1|n_{{}_{B}},n_{{}_{C}})=\frac{\alpha_{{}_{CB}}}{V}\cdot n_{{}_{B}}\cdot n_{{}_{C}},\;if\;\;\alpha_{{}_{CB}}<0$$

(27) $$T_{11}(n_{{}_{B}}+1|n_{{}_{B}},n_{{}_{C}})=\frac{\alpha_{{}_{CB}}}{V}\cdot n_{{}_{B}}\cdot n_{{}_{C}},\;if\;\;\alpha_{{}_{CB}}>0$$

(28) $$T_{12}(n_{{}_{C}}-1|n_{{}_{C}},n_{{}_{A}})=\frac{\alpha_{{}_{AC}}}{V}\cdot n_{{}_{C}}\cdot n_{{}_{A}},\;if\;\;\alpha_{{}_{AC}}<0$$

(29) $$T_{13}(n_{{}_{C}}+1|n_{{}_{C}},n_{{}_{A}})=\frac{\alpha_{{}_{AC}}}{V}\cdot n_{{}_{C}}\cdot n_{{}_{A}},\;if\;\;\alpha_{{}_{AC}}>0$$

(30) $$T_{14}(n_{{}_{C}}-1|n_{{}_{C}},n_{{}_{B}})=\frac{\alpha_{{}_{BC}}}{V}\cdot n_{{}_{C}}\cdot n_{{}_{B}},\;if\;\;\alpha_{{}_{BC}}<0$$

(31) $$T_{15}(n_{{}_{C}}+1|n_{{}_{C}},n_{{}_{B}})=\frac{\alpha_{{}_{BC}}}{V}\cdot n_{{}_{C}}\cdot n_{{}_{B}},\;if\;\;\alpha_{{}_{BC}}>0$$

(32) $$T_{16}(n_{{}_{C}}-1,n_{{}_{P}}+1|n_{{}_{C}},n_{{}_{P}})=\gamma \cdot n_{{}_{C}}$$

(33) $$T_{17}(n_{{}_{A}}-1|n_{{}_{A}})=\delta \cdot n_{{}_{A}}$$

(34) $$T_{18}(n_{{}_{B}}-1|n_{{}_{B}})=\delta \cdot n_{{}_{B}}$$

(35) $$T_{19}(n_{{}_{C}}-1|n_{{}_{C}})=\delta \cdot n_{{}_{C}}$$

(36) $$T_{20}(n_{{}_{C}}-1|n_{{}_{C}})=\xi \cdot n_{{}_{C}}$$

(37) $$T_{21}(n_{{}_{P}}-1|n_{{}_{P}})=\delta \cdot n_{{}_{P}}$$

(38) $$T_{22}(n_{{}_{C}}+1,n_{{}_{P}}-1|n_{{}_{C}},n_{{}_{P}})=\alpha_{res}e^{\beta t}\cdot n_{{}_{P}}$$

(39) $$T_{23}(n_{{}_{A}}-1|n_{{}_{A}})=\frac{\alpha_{{}_{AA}}}{V}\cdot n_{{}_{A}}\cdot (n_{{}_{A}}-1),\;if\;\;\alpha_{{}_{AA}}<0$$

(40) $$T_{24}(n_{{}_{A}}+1|n_{{}_{A}})=\frac{\alpha_{{}_{AA}}}{V}\cdot n_{{}_{A}}\cdot (n_{{}_{A}}-1),\;if\;\;\alpha_{{}_{AA}}>0$$

(41) $$T_{25}(n_{{}_{B}}-1|n_{{}_{B}})=\frac{\alpha_{{}_{BB}}}{V}\cdot n_{{}_{B}}\cdot (n_{{}_{B}}-1),\;if\;\;\alpha_{{}_{BB}}<0$$

(42) $$T_{26}(n_{{}_{B}}+1|n_{{}_{B}})=\frac{\alpha_{{}_{BB}}}{V}\cdot n_{{}_{B}}\cdot (n_{{}_{B}}-1),\;if\;\;\alpha_{{}_{BB}}>0$$

(43) $$T_{27}(n_{{}_{C}}-1|n_{{}_{C}})=\frac{\alpha_{{}_{CC}}}{V}\cdot n_{{}_{C}}\cdot (n_{{}_{C}}-1),\;if\;\;\alpha_{{}_{CC}}<0$$

(44) $$T_{28}(n_{{}_{C}}+1|n_{{}_{C}})=\frac{\alpha_{{}_{CC}}}{V}\cdot n_{{}_{C}}\cdot (n_{{}_{C}}-1),\;if\;\;\alpha_{{}_{CC}}>0$$

where, in the definition of each T($n_{i}\pm 1|n_{i}$), we included only those species actually taking part in the corresponding equation. Clearly, as in the deterministic formulation of the model, if any of the above mentioned *α* is equal to 0, no changes in the corresponding species will occur. Once the transition rates have been defined, we can define the master equation accounting for the probability of observing the system in its state **x** at time *t*, given the transition rates that describe the probability of passing from state **x** to state **y**. In its general form, the chemical master equation can be written as follows:

(45) $$\frac{d}{dt}P(\text{x},t)=\sum_{y\neq x}T(\text{x}|\text{y})P(\text{y},t)-T(\text{y}|\text{x})P(\text{x},t)$$

**Table 1:** Values of the model parameter together with their source.

Parameter	Description	Value	Unit	Reference
*μ*	Growth rate	0.7	*h*^−1^	[35]
*t*_*start*_, *t*_*end*_	Time of ABX exposure	12, 18	*h*	-
*δ*	Death rate	0.1	*h*^−1^	[36]
*γ*	Persisters generation rate	0 - 0.1	*h*^−1^	[21]
*ξ*	ABX killing rate	0 - 2.0	*h*^−1^	[37]
*α*_*res*_	Resuscitation rate	0.2	-	[25]
*ρ*	Growth defect of resuscitation rate	0.75	-	[25]

We will now introduce one step operator *ϵ* (or van Kampen operator) for each species in the model in order to be able to compact the formulation of the chemical master equation shown in Eq. (45).

(46) $$\epsilon_{{}_{A}}^{\pm }=f(n_{{}_{A}}\pm 1,n_{{}_{B}},n_{{}_{C}},n_{{}_{P}})$$

(47) $$\epsilon_{{}_{B}}^{\pm }=f(n_{{}_{A}},n_{{}_{B}}\pm 1,n_{{}_{C}},n_{{}_{P}})$$

(48) $$\epsilon_{{}_{C}}^{\pm }=f(n_{{}_{A}},n_{{}_{B}},n_{{}_{C}}\pm 1,n_{{}_{P}})$$

(49) $$\epsilon_{{}_{P}}^{\pm }=f(n_{{}_{A}},n_{{}_{B}},n_{{}_{C}},n_{{}_{P}}\pm 1)$$

where *f* is an arbitrary function that depends on the state **x**. The subscript of each step operator *ϵ* embeds in the pedix the information on which model species increases or decreases following the corresponding chemical reaction (e.g. $\epsilon_{A}$ for species *A*).

Accordingly, the explicit form of the chemical master equation governing the evolution of the discrete stochastic model assumes the following form:

$$\frac{d}{dt}P(\text{x},t)=[(\epsilon_{A}^{-}-1)T_{1}(n_{{}_{A}}+1|n_{{}_{A}})+(\epsilon_{B}^{-}-1)T_{2}(n_{{}_{B}}+1|n_{{}_{B}})+(\epsilon_{C}^{-}-1)T_{3}(n_{{}_{C}}+1|n_{{}_{C}})+(\epsilon_{A}^{+}-1)T_{4}(n_{{}_{A}}-1|n_{{}_{A}},n_{{}_{B}})+(\epsilon_{A}^{-}-1)T_{5}(n_{{}_{A}}+1|n_{{}_{A}},n_{{}_{B}})+(\epsilon_{A}^{+}-1)T_{6}(n_{{}_{A}}-1|n_{{}_{A}},n_{{}_{C}})+(\epsilon_{A}^{-}-1)T_{7}(n_{{}_{A}}+1|n_{{}_{A}},n_{{}_{C}})+(\epsilon_{B}^{+}-1)T_{8}(n_{{}_{B}}-1|n_{{}_{B}},n_{{}_{A}})+(\epsilon_{B}^{-}-1)T_{9}(n_{{}_{B}}+1|n_{{}_{B}},n_{{}_{A}})+(\epsilon_{B}^{+}-1)T_{10}(n_{{}_{B}}-1|n_{{}_{B}},n_{{}_{C}})+(\epsilon_{B}^{-}-1)T_{11}(n_{{}_{B}}+1|n_{{}_{B}},n_{{}_{C}})+(\epsilon_{C}^{+}-1)T_{12}(n_{{}_{C}}-1|n_{{}_{C}},n_{{}_{A}})+(\epsilon_{C}^{-}-1)T_{13}(n_{{}_{C}}+1|n_{{}_{C}},n_{{}_{A}})+(\epsilon_{C}^{+}-1)T_{14}(n_{{}_{C}}-1|n_{{}_{C}},n_{{}_{B}})+(\epsilon_{C}^{-}-1)T_{15}(n_{{}_{C}}+1|n_{{}_{C}},n_{{}_{B}})+(\epsilon_{C}^{+}\epsilon_{P}^{-}-1)T_{16}(n_{{}_{C}}-1,n_{{}_{P}}+1|n_{{}_{C}},n_{{}_{P}})+(\epsilon_{A}^{+}-1)T_{17}(n_{{}_{A}}-1|n_{{}_{A}})+(\epsilon_{B}^{+}-1)T_{18}(n_{{}_{B}}-1|n_{{}_{B}})+(\epsilon_{C}^{+}-1)T_{19}(n_{{}_{C}}-1|n_{{}_{C}})+(\epsilon_{C}^{+}-1)T_{20}(n_{{}_{C}}-1|n_{{}_{C}})+(\epsilon_{P}^{+}-1)T_{21}(n_{{}_{P}}-1|n_{{}_{P}})+(\epsilon_{C}^{-}\epsilon_{P}^{+}-1)T_{22}(n_{{}_{C}}+1,n_{{}_{P}}-1|n_{{}_{C}},n_{{}_{P}})+(\epsilon_{A}^{+}-1)T_{23}(n_{{}_{A}}-1|n_{{}_{A}})+(\epsilon_{A}^{-}-1)T_{24}(n_{{}_{A}}+1|n_{{}_{A}})+(\epsilon_{B}^{+}-1)T_{25}(n_{{}_{B}}-1|n_{{}_{B}})+(\epsilon_{B}^{-}-1)T_{26}(n_{{}_{B}}+1|n_{{}_{B}})+(\epsilon_{C}^{+}-1)T_{27}(n_{{}_{C}}-1|n_{{}_{C}})+(\epsilon_{C}^{-}-1)T_{28}(n_{{}_{C}}+1|n_{{}_{C}})]P(\text{x},t)$$

## 3 Results and discussion

### 3.1 The role of microbe-microbe interactions in persisters resuscitation

Microbial interactions can profoundly affect the dynamics of a microbial community, leading to very different end-points of each species [38]. We started by testing the effect of different interaction matrices on the final steady state of the community. We thus randomly sampled 100K interaction matrices in the interval [-1,1] and, for each of them, computed the end-point of the community. We found that, as expected [38], different interactions matrices led to different community end-points and to very different dynamics of each species in the model described by Eq.s (10)-(13) (Fig. 1F).

Among all the (randomly assembled) matrices, we identified and preliminary selected a matrix that could produce *i)* a stable steady state, *ii)* a community in which each species reached a non-zero final state and *iii)* where the pathogenic species showed the highest abundance at the end of simulations (Fig. 1B and C). Not surprisingly, this matrix was composed by negative interactions, as positive interactions are generally thought to be rare and to destabilise microbial communities [39], [40] (although recent evidence suggests that positive interactions may be less rare than we think [41]). Also, the values of these interaction matrices fall well in the range of other interaction matrices derived from other works [42], [1]. Finally, consistent with the underlying biology, the diagonal of this matrix is composed of negative entries as each of these species would eventually reach carrying capacity even in the absence of other species. Importantly, the final composition of the community did not change regardless of the initial abundance of each species (Fig. 1D). We then simulated the exposure to the antibiotic in the interval $t_{start}$ = 12 h and $t_{end}$ = 18 h. According to the model implementation (Fig. 1E), species *C* is affected by such perturbation and reaches a very low level of (relative) abundance. The other species react to such change by increasing (*A* and *B*) or decreasing (*P*) their abundance, as determined by the model structure. After antibiotic exposure, species *C* starts increasing again and the community gets to its new steady state. Using the end-points of such perturbed community as the initial points for a new simulation (without exposing it to the antibiotic) led to a final steady state identical to that of the initial simulation shown in Fig. 1C (See Figure S1). We next asked whether the structure of the community, i.e. the strength of the interactions among the three members, influenced the new steady state reached by species *C* following antibiotic exposure. We thus tested a wide range of possible interaction matrices in the interval [-1,0). As shown in Fig. 1F, the final composition of species *C* is profoundly affected by variations in the strength of the interactions among the members of the community. We analysed this aspect more thoroughly by sampling random interaction matrices in an increasingly narrower range and computing the final community composition for each of them (as the percentage of steady state communities including species *C*). We found that species *C* gets progressively excluded from the community with the increasing of the overall strength of microbe-microbe interactions (Fig. 1G). So, according to our model, the strength of the interactions of two other community members over a pathogenic, persisters-generating species may be enough to prevent the resuscitation of persisters following antibiotic treatment. This suggests that ecological interactions may play a role in determining the success of persisters resuscitation.

**Fig. 2:**
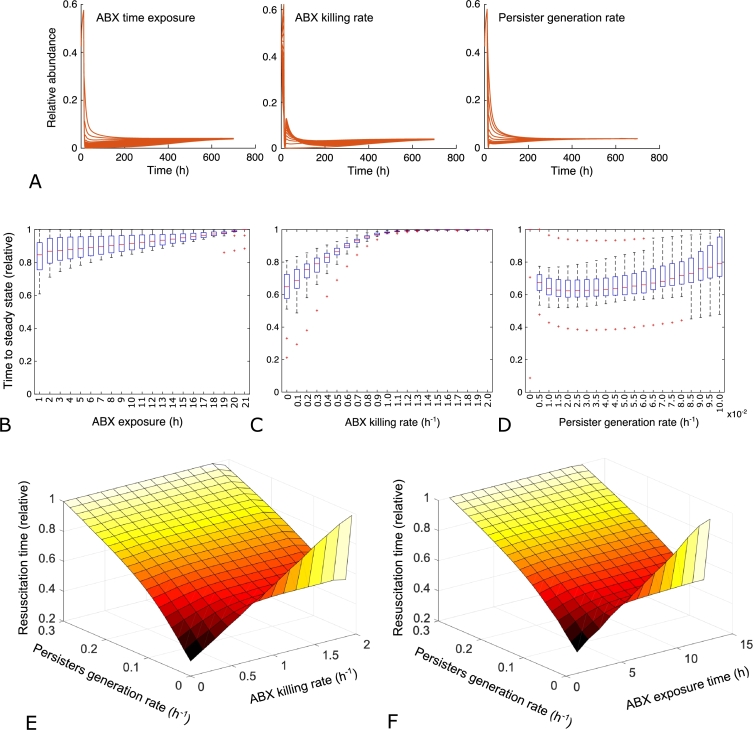
Changes in the main parameters of the model. A) Changes in antibiotic exposure time *t*_*end*_, antibiotic killing rate *ξ* and persister generation rate *γ* have no impact on the end-point values of species *C*. B-D) Impact on the post-antibiotic dynamic of species *C* of antibiotic exposure time (B), its strength (C) and persister generation rate (*γ*) (D). E) The effect of different antibiotic killing rate on the optimal resuscitation time. F) The effect of antibiotic exposure time on the optimal resuscitation time.

We then tested whether changes in the main parameters of the model (namely antibiotic exposure time $t_{end}$, antibiotic killing rate *ξ* and persister generation rate *γ*) could have any impact on the end-point values of species *C*. Furthermore, as we have previously shown that the interaction matrices can have a role in determining the final state of the corresponding community, we tested the impact of each parameter's value on a random sample of 1000 interaction matrices. As shown in Fig. 2A, changes in antibiotic time exposure, antibiotic killing rate and rate of persister formation did not significantly alter the final end-point of species *C*. However, what seemed to be affected was the so-called resuscitation time of persisters [22], [25], i.e. the time it takes for persister cells to start growing again after the antibiotic effect has vanished. Indeed, some differences among the effects of the tested parameters could be observed when considering the post-antibiotic dynamic of species *C* (Fig. 2A). To investigate this aspect more thoroughly, we measured the effect of the same three parameters on the time it took for the species *C* to get back to its steady state after exposing the community to the antibiotic. As shown in Fig. 2B and C, varying two of the three parameters led to expected consequences. Indeed, increasing the exposure time of the antibiotic (Fig. 2B) and its strength (Fig. 2C) is reflected in a progressively longer resuscitation time. When considering persisters generation rate, instead, we noticed a different trend: resuscitation time was maximal with *γ* equal to zero (no generation of persisters) and reached similarly high levels with a *γ* value of 0.1 $h^{-1}$. In between, we identified the most efficient response (i.e. the fastest of resuscitation time) with *γ* values ranging between 0.02 and 0.03 $h^{-1}$ (Fig. 2D). This suggests the presence of an optimum in persister generation rate, i.e. in the fraction of the population that is stochastically converted to persisting (dormant) cells. Higher values are not optimal likely because they reduce the population to extremely low values so that only persister cells are responsible for the resuscitation process and, as previously mentioned, their growth restart is likely to be impaired in respect to healthy cells [25]. Conversely, *γ* values that are too low to prevent the generation of enough persister cells to ensure the resuscitation after the exposure to the antibiotic treatment. Interestingly, among all the persisters generation rates tested, those that correspond to the apparently optimal resuscitation strategies fall in the range of the common persisters formation rate found in a wide range of microbial species [22].

Next, we asked whether the presence of an optimal resuscitation time was somehow linked to the values we chose for the other two parameters (*ξ* and $t_{end}$). Fig. 2E shows the effect of different antibiotic killing rate on the optimal resuscitation time. While we found that the presence of such an optimum was maintained regardless of the antibiotic killing rate, we also found that the optimal range narrows as the antibiotic killing rate increases. In other words, the stronger the killing power of the antibiotic, the more important it is to maintain the fraction of persister cells in the optimal range in order to provide a faster resuscitation time. While moving from this optimal range could have no effect (or even positive effects) in the presence of a weak antibiotic, doing so in the presence of a strong one may lead to significantly longer resuscitation times (Fig. 2E). We noticed the same trend also when examining the role of different *γ* values with varying antibiotic exposure time (Fig. 2F).

### 3.2 Accounting for noise in population dynamics affects persisters resuscitation

**Fig. 3:**
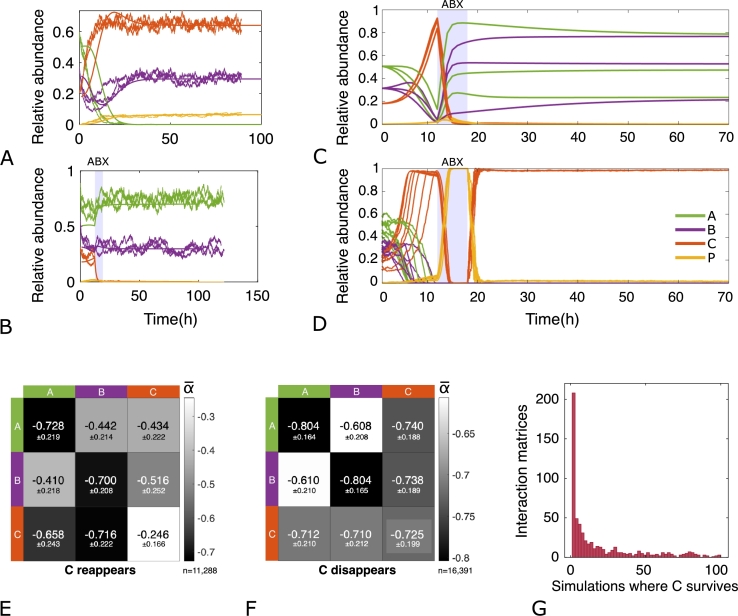
The effect of fluctuations in a three-member community. A-B) A sample of deterministic (straight line) and stochastic (step line) simulations. It is shown the overlap between the two models, both in a non-antibiotic (A) and antibiotic (B) scenario. However, stochastic effects may become relevant when the size of species *C* population reaches very low values. C) Deterministic simulation of nine communities using different interaction matrices that resulted in the extinction of species *C*. D) Using the same interaction matrices, species *C* reappears when modelling the community dynamics stochastically while *A* and *B* disappear from the community. Not all interaction matrices led to persister resuscitation, and thus to species *C* reappearance. Here we show in E) the average *α* values that lead to it (n=11,288 for each *α*), and in F) the average *α* values that lead species *C* to extinction even under the fluctuation system (n=16,391 for each *α*). In both matrices the standard deviation is reported. Furthermore, on G) we show the number of interaction matrices that led to the survival of species *C* when modelling the dynamics stochastically, and the number of simulations that used that specific matrix. In other words, there is a set of interaction coefficients that are more common than others (Species *A*: green, *B*: purple, *C*: red, *P*: yellow).

Up to now, we have modelled our antibiotic-perturbed community using a deterministic approach. Next, we simulated the same communities using a stochastic framework in which random fluctuations of species abundances are taken into account. A sample of such simulations is reported in Fig. 3, where we show that the stochastic simulations overlap with the deterministic ones both in a non-antibiotic and antibiotic scenario (Fig. 3A and B, respectively). While this agreement between stochastic and deterministic models was expected for large bacterial populations, when the size of species *C* population reaches very low values, stochastic effects may become relevant in determining *C* resuscitation. Thus, we reasoned that, when species *C* reaches very low population values following antibiotic exposure, stochasticity may begin to play an important role in determining the final end-point of the community. More specifically, we asked whether noise could impact the clearance of species *C* from the community when the deterministic model predicted so (i.e. a “no resuscitation” scenario). In other words, can random fluctuations in bacterial density counteract the effect of social interactions? In this regard, we specifically focused our attention on those simulation runs in which, following antibiotic exposure, the pathogen species *C* got extinct or approached very low abundance value (relative abundance lower than 1%). We explored 10^5^ different interaction matrices randomly sampled in the interval [-0.01,-1] and kept only those matrices that, following a 6 hours exposure to the antibiotic ($t_{start}$=12 and $t_{end}$=18), deterministically predicted a species *C* steady state with a relative abundance lower than 1%. These represent very interesting cases in which the structure of the community (i.e. the interactions among its members) are key to prevent the resuscitation of persisters. However, when dealing with low population sizes, stochastic fluctuations may lead to unpredictable steady states. In the present context, this may translate into a non zero probability of pathogen clearance and, in the worst case scenario, in the revival of the susceptible population. Let M be the set of such matrices (i.e. those that lead to the extinction of species *C* using the deterministic approach) and $n_{C}$ the number of cells of species *C*:

(50) $$M=\{M=[\alpha_{ij}]\;where\;i,j=A,B,C\;|\;n_{C}^{det}<1\text{\%}\}$$

We identified a total of 26,116 of such matrices and a snapshot of the deterministic dynamics of some of the underlying communities is reported in Fig. 3C. Then, to account for fluctuations in the number of cells for each species, we performed 100 simulations for each of the 26,116 interaction matrices using the stochastic rules described above. Each simulation had one interaction matrix and specific transition probabilities, i.e. the probability of each reaction to happen. In other words, when considering an infinitesimal time interval, we can assure that only one of the Eq. (17) - (44) will take place, accounting for the increase or decrease in *A*, *B*, *C* or *P* population by one cell. Once we had a simulation using one of the interaction matrices in M, we checked whether the stochastic end-points for the species *C* matched those predicted by the deterministic simulations. Interestingly, while in the vast majority of the simulations this turned out to be the case, we found instances in which the stochastic end-point of *C* was higher than that predicted by the deterministic model (Fig. 3D and C, respectively). Approximately, 1% of these simulations resulted in persisters' resuscitation and the survival of the antibiotic-sensitive species *C*. Let S be the subset of M that lead species *C* to survive using the stochastic framework:

(51) $$S=\{S\in M\;|\;n_{C}^{sto}>0\}$$

The average $\alpha_{ij}$ values in S showed that the self-interactions of *A* and *B* tend to be stronger than *C* self-interaction, i.e. ${\bar{\alpha }}_{A,A},\;{\bar{\alpha }}_{B,B}<{\bar{\alpha }}_{C,C}$, since they are negative interactions (Fig. 3E). On the other hand, average $\alpha_{ij}$ values in $S^{\text{c}}$, i.e. the interaction matrices that led species *C* to the extinction and to the overlap with the deterministic model, showed the opposite trend. Self-interaction of species *C* was stronger than the others, as well as the interactions between species *A* and *B*. In other words, there was an increase of ${\bar{\alpha }}_{C,C}$, ${\bar{\alpha }}_{A,B}$ and ${\bar{\alpha }}_{B,A}$ (Fig. 3F). These results highlight the importance of self-interactions in the fate of the perturbed species. From a biological stand point, since self-interactions account for the capability of a species to regulate its own abundance and/or a larger or smaller carrying capacity, this might underscore the importance of population-level features such as quorum sensing and/or nutrients exploitation in determining the relapse of species *C* in our community. However, S is not conformed by unique *S* matrices since there are several repeated elements, due to the different transition probabilities. In Fig. 3G we show the number of matrices that described the interactions of more than one simulation and let species *C* to survive. There were several interaction matrices *S* that described a small number of simulations where species C survived, and a small number of *S* that described a large number of them. The distribution of these matrices described how the interactions between the three members of the community have to be in order for species *C* to reappear, regardless the transition probabilities of each reaction. In Figure S2 we can see the *S* matrices that described the interactions of more than 94% of the communities simulated for each matrix, showing the $\alpha_{ij}$ values. In general, these matrices are characterised by a loose self interaction of species *C*, especially the ones that led *C* to survive 99% or 100% of the times, with $\alpha_{C,C}\approx 0$.

We next tested whether the state of the system, i.e. whether it has reached an equilibrium or not, may influence the dynamics of the entire community. Indeed, providing the antibiotic in the initial stages of species growth may result in the simultaneous application of two transient effects (antibiotic exposure and exponential growth of the species). We thus performed an additional set of simulations in which we allowed the system to get to the equilibrium (around t = 70 h) before adding the antibiotic (maintaining the exposure length as in previous simulations, i.e. 6 hours). Results of these simulations are shown in Figure S3 and were in good agreement with those shown in Fig. 3. However, one interesting features emerged. Postponing the antibiotic treatment to a time point where the species have reached an equilibrium point apparently reduces the probability of pathogen relapse in the community (see Figure S3A). Indeed, we found more instances in which the final community still embeds the pathogenic species in respect to simulations where the antibiotics was introduced from time t=12 to t=18 hours (see Figure S3E). Recent research has shown that early, aggressive, and sustained antibiotic regimens are often more effective at reducing relapse rates because they minimize the window of opportunity for persisters to repopulate. Infections involving pathogens like *Pseudomonas aeruginosa* demonstrate that improper timing of treatment can lead to the evolution of persister-enriched populations, complicating eradication efforts [43], [44].

## 4 Conclusions

In this work, we have assembled a simple theoretical community and studied the role of *i)* ecological interactions, *ii)* stress-related parameters and *iii)* stochastic fluctuations on the fate of persisters following a simulated antibiotic treatment. Our simulations indicated a key role of ecological interactions on the outcome of persisters once the stress (the antibiotics) vanished. While loose interactions generally resulted in the presence of the persisters-generating species in the final communities, tighter interactions concurred at the complete eradication of such species from the community. Interestingly, while antibiotic strength (efficacy) and treatment exposure correlated positively with persisters resuscitation, the rate at which persisters are formed displayed an optimum around the value commonly found in nature for this phenomenon. Finally, we specifically analysed those communities in which the ecological interactions concurred to the eradication of the pathogen using a stochastic framework that accounted for fluctuations at the level of cell counts. In this case, we observed that, in some cases, the pathogen species could resuscitate and eventually dominate the community following antibiotic treatment, a scenario that was never observed using the deterministic model. The relapse of bacterial infections due to persisters resuscitation is known to occur during real microbial infections eradication [25], [45], [16] and our simulations indicate that population size fluctuations might have a role therein and, more in general, in the resilience of microbial populations following a stressful perturbation. This work represents a first attempt to quantify the impact of the bacterial “social network” [46] on the survival and resuscitation of persisters in natural communities. In strictly clinical settings, the patterns we observed match our current knowledge derived from available experimental data. Overall, studies on antibiotic susceptibility that take microbial interaction networks and polymicrobial ecosystems into consideration are illuminating new bacterial survival strategies and therapeutic targets [47], including, for example, the observation that the persistence of one specific strain is negatively correlated with a close relative that shares the same metabolic necessities, i.e. both strains negatively interact one with the other [48], [49]. Other relevant cases in which the importance of microbial interactions on infection relapse has been shown experimentally include the interaction between the gut microbiota and *Clostridioides difficile* [50], [51], [52], *Pseudomonas aeruginosa* and *Stenotrophomonas maltophilia* in Cystic Fibrosis (CF) [53], *Helicobacter pylori* and *Lactobacillus* spp. in the stomach [54], [55] and polymicrobial infections of urinary tract [56], [57]. All these examples underscore the importance of providing a theoretical background to the dynamics of poly-microbial infections and on the consequences of perturbations therein. While we have obtained preliminary results that confirm the key role of microbe-microbe interactions in community dynamics, further work will be necessary to upscale our model to resemble real-world communities and/or to set up a mock community that resembles the one modelled here. Indeed, for example, we here assumed that the non-pathogenic members of the community (A and B) were not affected by the ABX treatment and this clearly biased the outcomes of our simulations. What if some of the interacting members of the community are also affected by the antibiotics (as it may happen with broad-range antibiotic treatments)? Further, it will be interesting to address the role of ecological interactions in persisters resuscitation when the size of the community (including the number of pathogenic, persister-generating species) increases. Indeed, species richness and the strength of species interactions have been shown to regulate community dynamics [58]. Moreover, varying the antibiotic treatment strategy in a more drastic way could produce different outcomes from those that we obtained here. Antibiotics with a broad spectrum of action (or the use of multiple antibiotics) and/or the manipulation of antibiotic killing time have been shown to induce different responses in the target microbial community [59], [60]. Similarly, stochastic modelling has shown that the scenario of density regulation and the mode of action of the drug influence the survival of a resistant subpopulation [33]. Would these features hold true also when ecological interactions and stochastic fluctuations are considered at the same time? Finally, the modelling framework developed here is general enough to be used, in the future, for the study of microbial communities dynamics in other contexts in which the anthropogenic impact, among the others, is known to cause significant alterations to the community profile. These could include, for example, waste water treatment plants [61], the gut microbiota [62], [63], agroecosystems [64] and natural systems in general [64].

## Material and methods

All the simulations were carried out using MATLAB v. 2022b. All the codes used in this work are available at Zenodo (https://doi.org/10.5281/zenodo.12598803).

## CRediT authorship contribution statement

**Tania Alonso-Vásquez:** Writing – review & editing, Validation, Conceptualization. **Michele Giovannini:** Writing – review & editing, Conceptualization. **Gian Luigi Garbini:** Writing – review & editing, Conceptualization. **Mikolaj Dziurzynski:** Writing – review & editing, Conceptualization. **Giovanni Bacci:** Writing – review & editing, Conceptualization. **Ester Coppini:** Supervision, Funding acquisition. **Donatella Fibbi:** Supervision, Funding acquisition. **Marco Fondi:** Writing – review & editing, Writing – original draft, Methodology, Investigation, Conceptualization.

## Declaration of Competing Interest

The authors declare the following financial interests/personal relationships which may be considered as potential competing interests: Marco Fondi reports financial support was provided by Italian Ministry of University and Research (Grant code: 2022Z88RK4). If there are other authors, they declare that they have no known competing financial interests or personal relationships that could have appeared to influence the work reported in this paper.

## Appendix A Supplementary material

MMC Figures to describe the dynamics of a three-member community in three different situations: 1. Steady-state points as initial conditions. 2. Interaction matrices where the pathogen species survives. 3. ABX treatment at a steady-state situation. MMC
