# Supplementary material for: An ecological and stochastic perspective on persisters resuscitation
Source: Comput Struct Biotechnol J. 2024 Dec 6;27:1–9. doi: 10.1016/j.csbj.2024.12.002 (PMC11697298; doi:10.1016/j.csbj.2024.12.002)
Supplement: MMC — Figures to describe the dynamics of a three-member community in three different situations: 1. Steady-state points as initial conditions. 2. Interaction matrices where the pathogen species survives. 3. ABX treatment at a steady-state situation. [file mmc1.docx]

**An ecological and stochastic perspective on persisters resuscitation**

Tania Alonso-Vásquez^a^, Michele Giovannini^a^, Gian Luigi Garbini^a^, Mikolaj Dziurzynski^a^, Giovanni Bacci^a^, Ester Coppini^b^, Donatella Fibbi^b^, Marco Fondi^a^

^a^Department of Biology, University of Florence, Via Madonna del Piano 6, Sesto Fiorentino, 50019, Italy, Italy

^b^G.I.D.A. SpA, Via Baciacavallo 36, Prato, 59100, Italy

**Supplementary material**

***Figure S1:*** Dynamics of the three-member community of Figure 1E, but with the end-points of such perturbed community as the initial points for a new simulation without exposing it to the antibiotic. Here we show that different initial points lead to a final steady state the matched that of the initial simulation shown in Figure 1C, the community without antibiotic perturbations.

***Figure S2:*** These interaction matrices are elements of *S*, and are represented on the right side of the distribution in Figure 3G. When these α values are used to describe the interactions between the members of 100 different communities, species C survives 100%, 99%, 98%, 97% and 95% of the times, respectively (i.e. number of C cells *n_C_* is greater than 0).

***Figure S3:*** Dynamics of a three-member community when the antibiotic treatment occurs on a steady-state situations (t*_start_*=70h, t*_end_*=18h). Using the same interaction matrices, here we show the deterministic (A) and the stochastic (B) simulations. E) The average α values that lead to species *C* reappearance and D) the average α values that lead to species *C* to extinction even under the fluctuations of the system. G) The number of interaction matrices that led to the survival of species *C* when modelling the dynamics stochastically, and the number of simulations that used that specific matrix. In other words, there is a set of interaction coefficients that are more common than others. (Species *A*: green, *B*: purple, *C*: red, *P*: yellow).
